# Life events impact on experiences of COVID-19 pandemic (in Azerbaijani sample)

**DOI:** 10.1192/j.eurpsy.2022.1241

**Published:** 2022-09-01

**Authors:** E. Ismailova, A. Ryzhov, E. Zhuykova, L. Pechnikova, E. Sokolova

**Affiliations:** 1 Lomonosov MSU, Baku Branch, Baku, Azerbaijan; 2 Lomonosov MSU, Faculty Of Psychology, Moscow, Russian Federation; 3 Russian State University for the Humanities, L.s. Vygotsky Institute Of Psychology, Moscow, Russian Federation

**Keywords:** life events, timeline, counseling, Covid-19

## Abstract

**Introduction:**

Many studies point to cognitive beliefs, attitudes and other psychologicalt traits involved in particularities of reactions to pandemic situation, but the differences in life events are often overlooked.

**Objectives:**

A study of subjective evaluation of life events during the pandemics.

**Methods:**

The modified Lifeline technique was used to elicit life events. In semistructured interview, using a timeline, subjects were asked to indicate and describe events that had an impact on their attitudes, behaviors and feelings since the start of pandemic. Then they evaluated with direct assessment scales each event as to what extent it was anxious, difficult to cope, changed the beliefs concerning COVID-19, fostered the changes of behavior and habits, and led to reappraisal of own values. The events were coded using dichotomous categories: COVID-related vs directly unrelated, universal vs individual, personally involved vs noninvolved, and also were further qualitatively evaluated. 25 young Azerbaijani residents took part in the study.

**Results:**

From 191 events named, 72% were COVID-related, 62% - universal, 62% - with personal involvement. 46% of events were unique (mentioned once). Universal events were more likely to be assessed as anxiogenic, while personal ones as leading to rethink own values and priorities (U, p<.01 and p<.05). Surprisingly, life events in total were assessed as less challenging the beliefs about pandemics while more frequently leading to rethink own values (T, p<.05). Individual events involved more conflict meanings and implications.

**Conclusions:**

Lifeline technique may provide important insights on the impact of life events in complex social transitions and may be used in counseling.

**Disclosure:**

No significant relationships.

